# Mapping of Notch signaling in the developing organ of Corti in common marmosets

**DOI:** 10.3389/fnana.2023.1188886

**Published:** 2023-06-07

**Authors:** Makoto Hosoya, Masato Fujioka, Hideyuki Okano, Hiroyuki Ozawa

**Affiliations:** ^1^Department of Otolaryngology, Head and Neck Surgery, Keio University School of Medicine, Shinjuku City, Japan; ^2^Department of Molecular Genetics, Kitasato University School of Medicine, Sagamihara, Japan; ^3^Department of Physiology, Keio University School of Medicine, Shinjuku City, Japan; ^4^Laboratory for Marmoset Neural Architecture, Center for Brain Science, Saitama, Japan

**Keywords:** cochlea, marmoset, Notch, cochlear development, inner ear

## Abstract

**Introduction:**

The well-regulated development of the sensory epithelium is essential for hearing. This process involves the specification of a pro-sensory epithelium containing common progenitors that differentiate into hair and supporting cells. Notch signaling is one of the most critical pathways during these processes, and its modification is thought to be a feasible approach for treating hearing loss. Despite interspecies differences between rodents and primates or humans, most of our current knowledge regarding cochlear development has been obtained from rodent models.

**Methods:**

We therefore examined and mapped the expression patterns of Notch signal components in the developing cochlea of the common marmoset (*Callithrix jacchus*), a small monkey species native to the New World, a primate model animal.

**Results:**

In contrast to the preserved expression patterns of the Notch signaling components in the hair cell differentiation between primates and rodents, we unveiled relatively large interspecies differences during the maturation of supporting cells.

**Discussion:**

This improved knowledge of Notch signaling during primate cochlear development will facilitate the development of future regenerative therapies.

## 1. Introduction

Hearing is the process by which mechanical sound waves are sensed in the inner ear, converted into electrical impulses in the spiral ganglion neurons, and perceived by the brain. The cochlea and its organ of Corti, which consists mainly of inner hair cells, outer hair cells, and supporting cells, play an essential role in these processes. More specifically, hair cells in the cochlea convert mechanosensory sound waves into neural electrical pulses. This mechano-electrical conversion is structurally and electrically facilitated by supporting cells surrounding the hair cells. These electrical pulses are then transmitted through synapses between the inner hair cells and spiral ganglion neurons. Eventually, they reach the auditory cortex of the brain, where they are perceived as hearing sensation (Stover and Diensthuber, 2011; Ekdale, 2016).

The well-regulated development of the sensory epithelium is essential for hearing (Wu and Kelley, 2012; Roccio et al., 2020). This process involves the specification of a pro-sensory epithelium containing common progenitors of hair and supporting cells, followed by their differentiation into hair and supporting cells. These processes have been reported to be regulated by signaling pathways, such as Notch (Morrison et al., 1999; Eddison et al., 2000; Kiernan et al., 2001; Kiernan, 2013), Wnt (Ladher et al., 2000; Wu and Kelley, 2012), Shh (Riccomagno et al., 2002, 2005), and Fgf (Mckay et al., 1996; Ladher et al., 2000) signalings, which are also involved in the organogenesis of other tissues.

During cochlear development, Notch signaling is involved in the specification of the sensory epithelium and the subsequent differentiation of hair or supporting cells, as well as in the development of spiral ganglion neurons (Fekete and Wu, 2002; Groves and Fekete, 2012; Murata et al., 2012). To date, studies have revealed that, in the premature cochlea, “Notch-on” cells differentiate into the pro-sensory epithelium in a “lateral induction” manner. During late cochlear development, “Notch-off” cells in the differentiated pro-sensory epithelia differentiate into hair cells, whereas “Notch-on” cells differentiate into supporting cells in a “lateral inhibition” manner (Kiernan, 2013).

Extensive knowledge about the role of Notch signaling in cochlear development has been obtained using rodents, particularly mice, as model animals. Recently, however, primate model animals, such as the common marmoset, *Callithrix jacchus*, have been used, and several interspecies differences have been reported in cochlear development between rodents and primates (Hosoya et al., 2021a,b,c, 2022). In particular, several significant interspecies differences have been reported in the gene expression patterns of supporting cells. For example, the expression patterns of cyclin-dependent kinase inhibitor 1B (CDKN1B), GATA3, and ISL1 in developing supporting cells differ between rodents and primates (Hosoya et al., 2020). Moreover, differences have been reported in the initial expression pattern of JAG1, which is a well-defined Notch ligand, between mice and primates (Hosoya et al., 2022). In addition, specific differences also exist in the tempo of cochlear development between rodents and primates (Hosoya et al., 2021b,^2022^), and the impact of these differences on cochlear development cannot be underestimated (Hosoya et al., 2021b).

Therefore, based on previous observations in primate cochlear models (Hosoya et al., 2016, 2021b,c,^2022^), we hypothesized the existence of interspecies differences in the activation of Notch signaling and expression patterns of its components between primates and rodents. We used the common marmoset, a primate model animal, to test this hypothesis.

## 2. Materials and methods

### 2.1. Specimens

Cadaverous temporal bone samples of common marmosets at E77 (*n* = 2), E87 (*n* = 4), E92 (*n* = 3), E96 (*n* = 5), E101 (*n* = 4), E109 (*n* = 3), E115 (*n* = 4), E120 (*n* = 3), and P0 (*n* = 5) were used in this study. All animal experiments were approved by the Animal Experiment Committee of Keio University (approval numbers: 11006 and 08020) and were performed in accordance with the guidelines of the National Institutes of Health and the Ministry of Education, Culture, Sports, Science, and Technology of Japan.

### 2.2. Tissue preparation

Immediately after euthanasia, the temporal bone was dissected and fixed with 4% paraformaldehyde in phosphate-buffered saline (PBS) overnight. P0 specimens were decalcified in Decalcifying Solution B (Wako, Osaka, Japan) for 1 week. Specimens were embedded in Tissue-Tek O.C.T. compound (Sakura Finetechnical Co., Ltd., Tokyo, Japan) for cross-sectioning, and 7-μm sections were used for immunohistochemical analysis.

### 2.3. Antibodies

The following primary antibodies were used: anti-NOTCH1 (1:500, rabbit IgG, 3608, Cell Signaling Technology, Danvers, MA, USA), anti-HES1 (1:200, rabbit IgG, 11988, Cell Signaling Technology), anti-RBPJ (1:1000, rabbit IgG, 5313, Cell Signaling Technology), anti-JAG1 (1:200, rabbit IgG, ab109536, Abcam, Cambridge, UK), anti-DLL1 (1:100, mouse IgG2b, MAB18181, R&D, Minneapolis, MN, USA), anti-POU4F3 (1:200, mouse IgG1, sc-81980, Santa Cruz Biotechnology, Santa Cruz, CA, USA), anti-ATOH1 (1:500, rabbit IgG, 21215-1, Proteintech, Rosemont, IL, USA), anti-SOX2 (1:200, goat IgG, AF2018, R&D), anti-MYOSIN7A (1:30, mouse IgG1, 138-1-s, DSHB, Iowa City, IA, USA), anti-MYOSIN7A (1:100, rabbit IgG, 25-6790, Proteus Biosciences, Ramona, CA, USA), anti-CDKN1B (1:200, mouse IgG1, 610242, BD, Franklin Lakes, NJ, USA). The following secondary antibodies were used: donkey anti-rabbit IgG, Alexa Fluor Plus 555 (1:500, A32794, Invitrogen), donkey anti-mouse IgG, Alexa Fluor Plus 488 (1:500, A32766, Invitrogen), and donkey anti-goat IgG, Alexa Fluor 647 (1:500, 705-605-147, Jackson Immuno-Research).

### 2.4. Immunohistochemistry

After a brief wash with PBS, sections were heated (80°C) in 10 μM citrate buffer (pH 6) for 15 min. Following another brief wash, sections were blocked for 1 h at room temperature in 10% normal serum in PBS, incubated overnight with primary antibodies at 4°C, and then incubated with Alexa Fluor-conjugated secondary antibodies for 60 min at room temperature. For immunostaining with NOTCH1, HES1, and RBPJ antibodies, sections were incubated in 10% normal serum in PBS supplemented with 0.01% Triton-X. Nuclei were counterstained with Hoechst 33258.

## 3. Results

### 3.1. Nuclear localization of NOTCH1 in the developing cochlea

**FIGURE 1:**
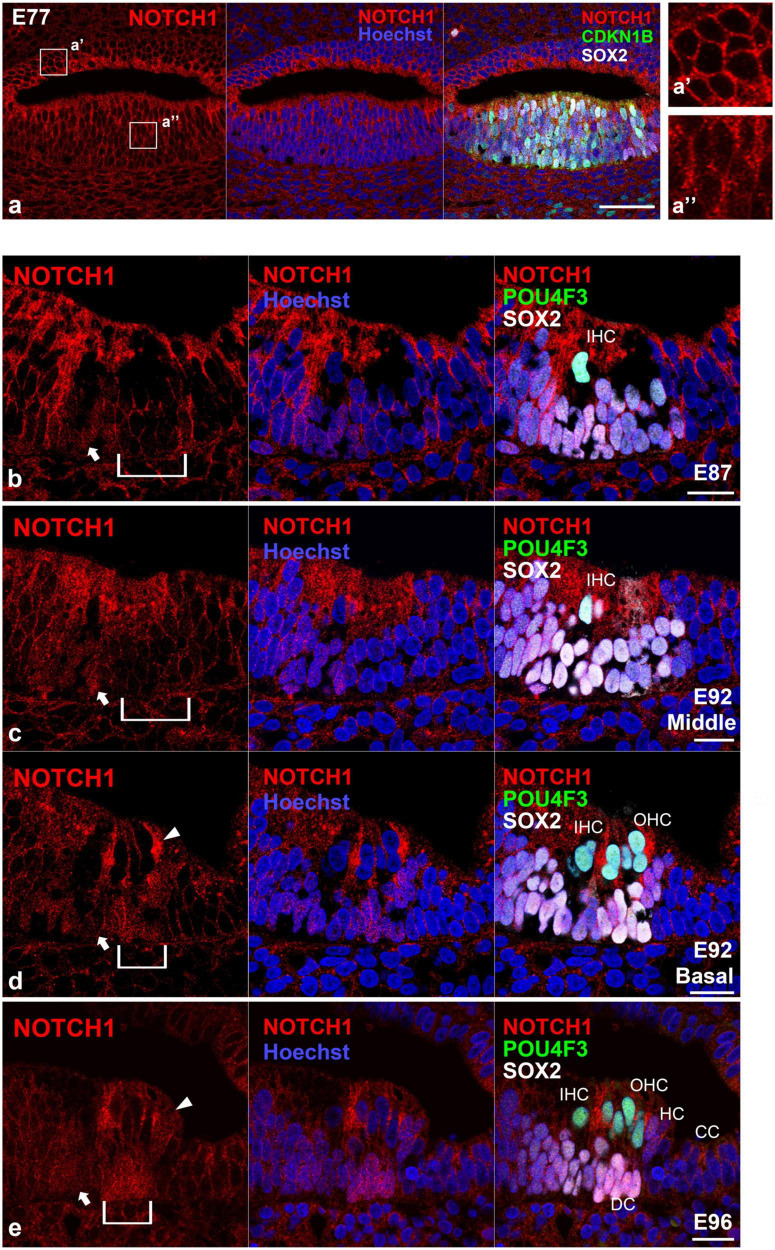
Intranuclear localization of NOTCH1 in the early developing cochlea. **(a)** In E70 cochlea, weak intranuclear localization of NOTCH1 was detected in the CDKN1B- and SOX2- positive pro-sensory epithelium (a’), whereas no intranuclear localization of NOTCH1 was detected in the ventral cochlear duct (a”). **(b)** In the E87 cochlea, intranuclear localization of NOTCH1 was observed in cells surrounding inner hair cells in the organ of Corti (arrow). Conversely, no expression was detected in the undifferentiated outer hair cell region (bracket). **(c,d)** In the E92 cochlea, in the middle turn, nuclear localization of NOTCH1 was observed only in supporting cells surrounding inner hair cells [arrow in **(c)**]. In contrast, no expression was detected in the undifferentiated outer hair cell region [bracket in **(c)**]. In the basal turn, intranuclear localization of NOTCH1 was observed in supporting cells surrounding inner hair cells [arrow in **(d)**] and cells surrounding outer hair cells [bracket in **(d)**]. Intranuclear localization was also observed in Hensen’s cells [arrowhead in **(d)**]. **(e)** In the E96 cochlea, nuclear localization of NOTCH1 was observed in supporting cells [arrow and bracket in **(e)**], including Deiters’ and Hensen’s cells (arrowhead), except Claudius’ cells. Nuclei were counterstained with Hoechst (blue). Scale bar: 50 μm in **(a)**, 20 μm in **(b–e)**. IHC, inner hair cell; OHC, outer hair cell; HC, Hensen’s cell; DC, Deiters’ cell; CC, Claudius’ cell. **(b)**: basal turn, **(e)**: middle turn.

**FIGURE 2:**
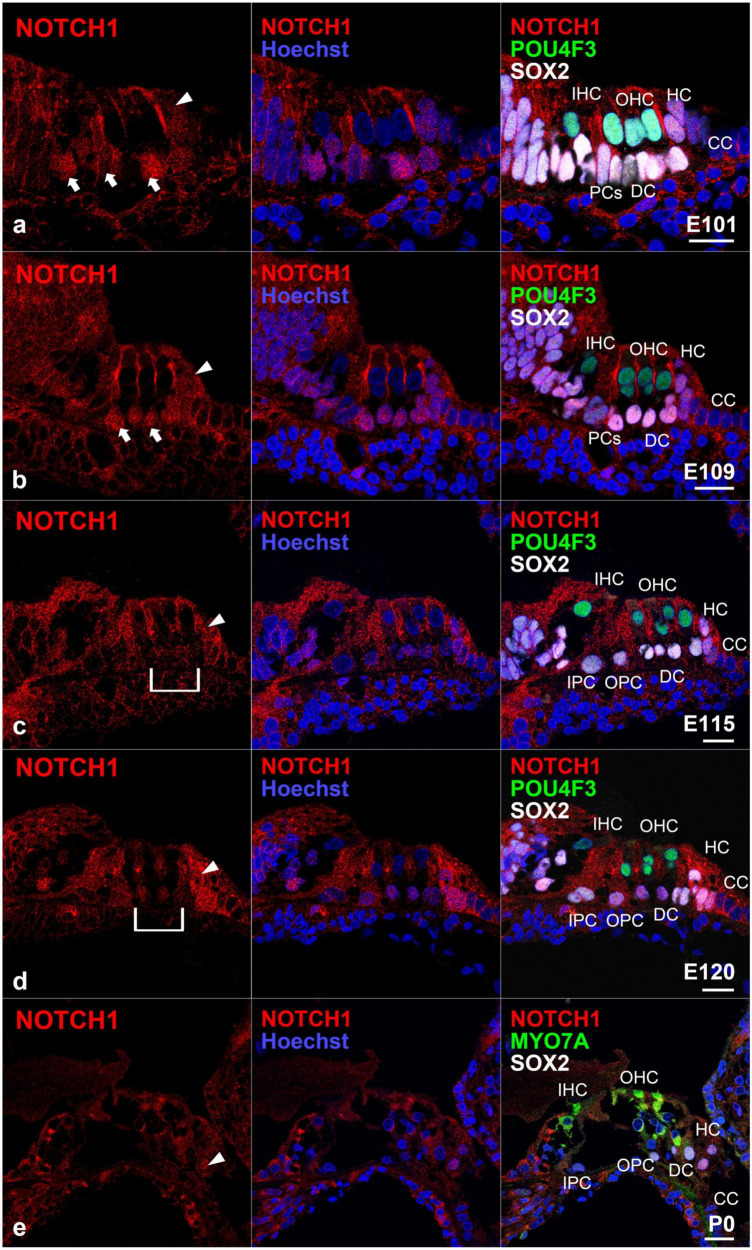
Intranuclear localization of NOTCH1 in the late-developing cochlea. **(a)** In E101 cochlea, nuclear localization of NOTCH1 was observed in supporting cells (arrows), including Hensen’s cells (arrowhead) but not Claudius’ cells. **(b)** In the E109 cochlea, nuclear localization of NOTCH1 was observed in supporting cells (arrows), including Hensen’s cells (arrowhead) but not Claudius’ cells. **(c)** In the E115 cochlea, the nuclear localization of NOTCH1 in Deiters’ cells [bracket in **(c)**] was decreased, whereas remained robust in Hensen’s cells (arrowhead) and was still detected in pillar cells. **(d)** In E120 cochlea, the nuclear localization of NOTCH1 in Deiters’ cells [bracket in **(c)**] was relatively low compared with that in Hensen’s (arrowhead), inner pillar, and outer pillar cells. **(e)** In the P0 cochlea, nuclear localization of NOTCH1 was observed only in Hensen’s cells (arrowhead) in the organ of Corti. Nuclei were counterstained with Hoechst (blue). Scale bar: 20 μm. IHC, inner hair cell; OHC, outer hair cell; HC, Hensen’s cell; DC, Deiters’ cell; CC, Claudius’ cell’ PCs, pillar cells; IPC, inner pillar cells; OPC, outer pillar cells. **(a,d)**: middle turn, **(b,c,e)**: basal turn.

In this study, we first examined the nuclear localization of NOTCH1 (Figures 1, 2). The NOTCH1 antibody used in this study recognizes the transmembrane domain of NOTCH1 and NICD and thus can detect the nuclear localization of NICD, which results in the activation of Notch signaling (Troy et al., 2013; Shimizu et al., 2021). We observed a relatively weak nuclear localization of NOTCH1 in SOX2- and CDKN1B-positive sensory epithelia but not in non-sensory epithelia in the cochlea of E77 common marmosets (Figure 1a). However, we observed the nuclear localization of NOTCH1 in cells surrounding the inner hair cells in the organ of Corti in the E87 cochlea (Figure 1b). Notably, we detected the nuclear localization of NOTCH1 in both supporting cells surrounding inner hair cells and cells surrounding outer hair cells in the basal turn in the E92 cochlea (Figures 1c, d). However, in the middle turn, in which outer hair cells have not been differentiated, we observed the nuclear localization of NOTCH1 only in supporting cells surrounding inner hair cells. Apart from Claudius’ cells, we detected the nuclear localization of NOTCH1 in supporting cells in the cochlea of E96 (Figure 1e) up to E109 (Figures 2a, b) common marmosets. We found that the nuclear localization of NOTCH1 was decreased in Deiters’ cells, whereas we still observed a robust NOTCH1 nuclear localization in Hensen’s cells in E115 and E120 cochlea (Figures 2c, d). However, NOTCH1 was localized only in Hensen’s cell nuclei in the organ of Corti in the P0 cochlea (Figure 2e).

### 3.2. Expression of HES1 in the developing organ of Corti

**FIGURE 3:**
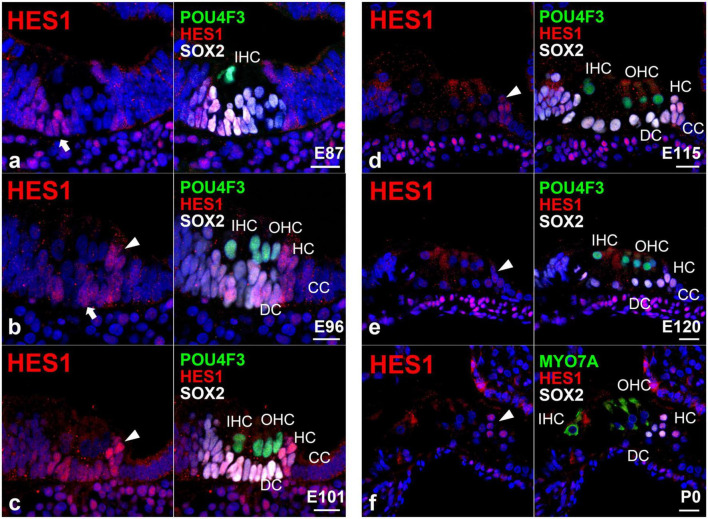
Expression pattern of HES in the developing cochlea of primates. **(a)** In E87 cochlea, HES1 was expressed in cells surrounding inner hair cells in the organ of Corti (arrow). In contrast, no expression was detected in the undifferentiated outer hair cell region. **(b)** In the E96 cochlea, HES1 was expressed in both supporting cells surrounding inner and outer hair cells [arrow in **(b)**]. HES1 was also expressed in Hensen’s cells [arrowhead in **(b)**]. **(c)** In the E101 cochlea, except Claudius’ cells, HES1 was expressed in supporting cells, including Deiters’ and Hensen’s cells (arrowhead). **(d,e)** After E115, the expression of HES1 was gradually decreased in supporting cells except for Hensen’s cells [arrowhead in **(e)**]. **(f)** In the P0 organ of Corti, HES1 was expressed only in Hensen’s cells. Nuclei were counterstained with Hoechst (blue). Scale bar: 20 μm. IHC, inner hair cell; OHC, outer hair cell; HC, Hensen’s cell; DC, Deiters’ cell; CC, Claudius’ cell. **(a,f)**: basal turn, **(b–e)**; middle turn.

Next, we investigated the expression pattern of hairy and enhancer of split 1 (HES1) in the developing organ of Corti (Figure 3). HES1 is a bHLH transcription factor encoded by *HES1* and is the mammalian homolog of the *hairy* gene in *Drosophila* (Feder et al., 1994). *HES1* is one of the seven members of the *HES* gene family (*HES1-7*), which encode nuclear proteins that suppress transcription. During inner ear development, HES1 acts as a downstream effector of the Notch signaling pathway and suppresses the differentiation of the pro-sensory epithelium into hair cells by repressing the expression of *ATOH1* (Zine and de Ribaupierre, 2002).

In this study, we detected the expression of HES1 in supporting cells surrounding the inner hair cells in the E87 cochlea (Figure 3a). We also observed that HES1 was expressed in supporting cells in E96 and E101 cochlea (Figures 3b, c). However, in E115 and E120 cochlea, the expression of HES1 was decreased in Deiters’ cells, whereas it was still detected in Hensen’s cells (Figures 3d, e). Finally, we detected that HES1 was expressed only in Hensen’s cells in the P0 organ of Corti (Figure 3f). Collectively, we demonstrated that the changes in the expression patterns of HES1 in the developing organ of Corti paralleled the changes in the activation of NOTCH1. This observation suggested that during primate cochlear development, the expression of HES1 is controlled by the activation of NOTCH1 as previously reported in rodents.

### 3.3. Expression of RBPJ in the developing organ of Corti

**FIGURE 4:**
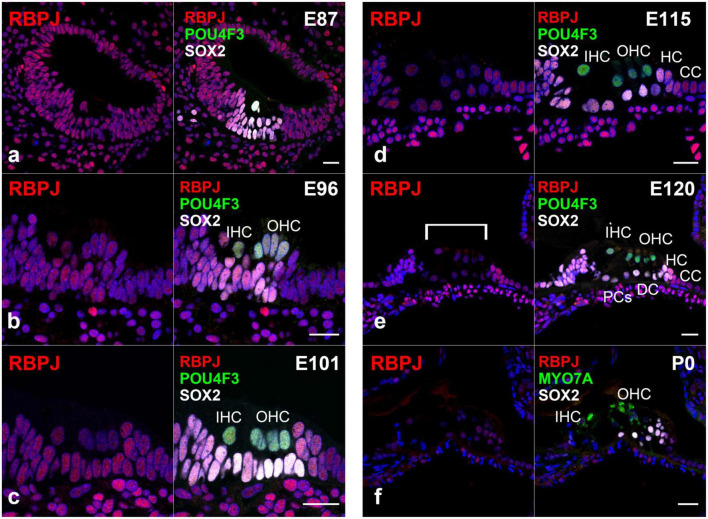
Expression pattern of RBPJ in the developing cochlea of primate. **(a)** In the E87 cochlea, RBPJ was ubiquitously expressed in the cochlear duct and surrounding cells. **(b–d)** In E96, E101, and E115 cochlea, RBPJ was ubiquitously expressed in the organ of Corti. **(e)** Likewise, RBPJ was ubiquitously expressed in the organ of Corti in E120 cochlea. However, the expression of RBPJ in inner hair, outer hair, pillar, and Deiters’ cells was relatively low (bracket) compared with that in other cells, such as Hensen’s and Claudius’ cells. **(f)** In the P0 organ of Corti, the expression of RBPJ was ubiquitously diminished, and only a slight expression was observed. Nuclei were counterstained with Hoechst (blue). Scale bar: 20 μm. IHC, inner hair cell; OHC, outer hair cell; HC, Hensen’s cell; DC, Deiters’ cell; CC, Claudius’ cell; PCs, pillar cells. **(a)**: basal turn, **(b–e)**; middle turn, f; apical turn.

**FIGURE 5:**
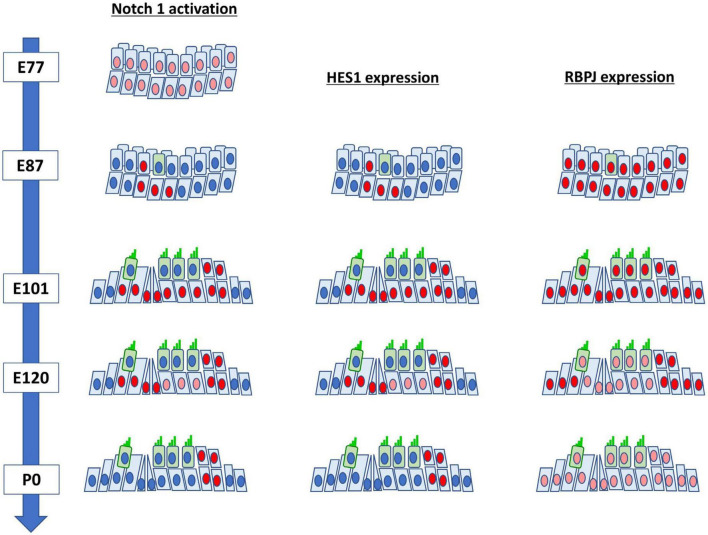
Schematic diagrams of the activation of NOTCH1 and expression patterns of HES1 and RBPJ in the developing cochlea of the common marmoset.

Next, we investigated the expression pattern of RBPJ in the developing organ of Corti (Figure 4). We observed the ubiquitous expression of RBPJ in the cochlea epithelium in the E87 cochlea (Figure 4a). We also observed that RBPJ was expressed in inner and outer hair cells and supporting cells up to E115 cochlea (Figures 4b–d). However, after E120, we found that the expression of RBPJ in hair, pillar, and Deiters’ cells was decreased, whereas a relatively strong expression was still observed in other supporting cells (Figure 4e). We found that RBPJ was still expressed in the P0 organ of Corti, despite its downregulation compared with the expression levels observed in early developmental stages (Figure 4f). Schematic diagrams of the activation of NOTCH1 and expression patterns of HES1 and RBPJ are depicted in Figure 5.

### 3.4. Expression of JAG1 and DLL1 in the developing organ of Corti

**FIGURE 6:**
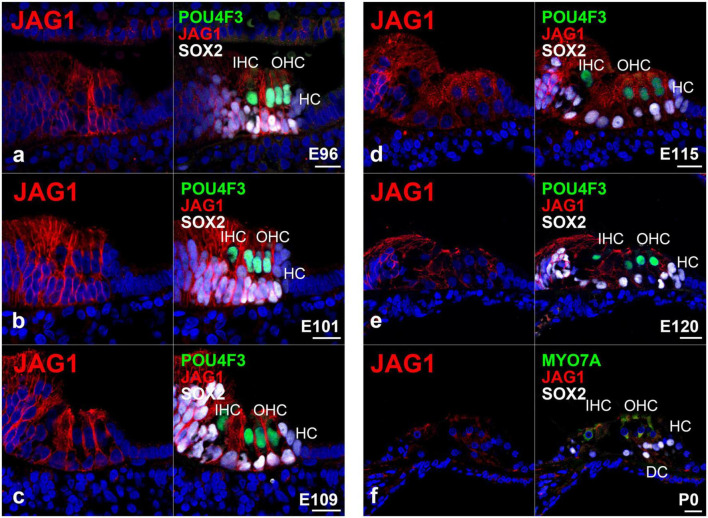
Expression pattern of JAG1 in the developing cochlea of primates. **(a)** In E96 cochlea, JAG1 was expressed in both hair cells and SOX2-positive supporting cells, except immature Hensen’s cells. **(b–d)** In E101, E109, and E115 cochlea, JAG1 was expressed in both hair cells and SOX2-positive supporting cells, except immature Hensen’s cells similar to E92 cochlea. **(e)** After E120, the expression of JAG1 in the organ of Corti gradually decreased. **(f)** In the P0 organ of Corti, a weak expression of JAG1 was only observed in Deiters’ cells. Nuclei were counterstained with Hoechst (blue). Scale bar: 20 μm. IHC, inner hair cell; OHC, outer hair cell; HC, Hensen’s cell; DC, Deiters’ cell. **(a,c,f)**: basal turn, **(b,d,e)**: middle turn.

**FIGURE 7:**
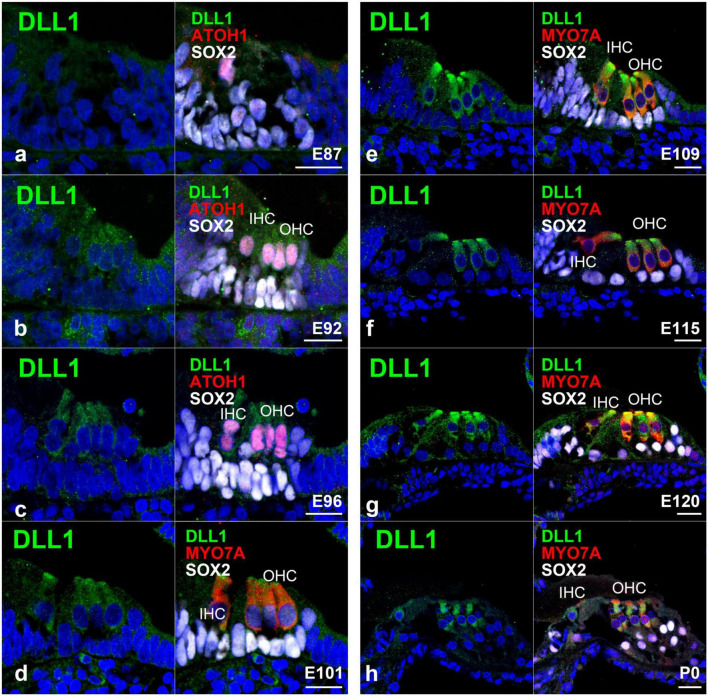
Expression pattern of DLL1 in the developing cochlea of primates. **(a)** In E87 cochlea, no expression of DLL1 was detected in either ATOH1-positive hair cells or SOX2-positive supporting cells. **(b)** In contrast, a relatively weak expression of DLL1 was observed in ATOH1-positive hair cells in the E92 cochlea. **(c)** In E96 cochlea, the expression of DLL1 in hair cells became more pronounced. **(d–h)** After E101, DLL1 was continuously expressed in hair cells. Nuclei were counterstained with Hoechst (blue). Scale bar: 20 μm. IHC, inner hair cell; OHC, the outer hair cell. **(a,b,d,g,h)**: basal turn, **(c,e,f)**: middle turn.

Finally, we investigated the expression pattern of jagged canonical Notch ligand 1 (JAG1) (Figure 6) and delta-like canonical Notch ligand 1 (DLL1) (Figure 7) in the developing organ of Corti. JAG1 and DLL1 are 2 of 5 ligands interacting with Notch receptors. Once JAG1 (or DLL1) interacts with NOTCH, a proteolytic cleavage cascade is triggered, resulting in the nuclear translocation of NICD and the subsequent transcriptional activation of downstream target genes. *Jag1* and *Dll1* are reportedly essential for the normal development of hair cells (Brooker et al., 2006).

Previously, we reported the expression pattern of JAG1 in the E77, E87, and E92 cochlea of the common marmoset (Hosoya et al., 2022). Interestingly, we observed that JAG1 was expressed in the cochlea of the common marmoset after the E87 stage. Therefore, in this study, we examined the expression pattern of JAG1 in the cochlea after the E96 stage. We detected the expression of JAG1 in SOX-2 positive supporting cells except for Hensen’s cells in the E96 organ of Corti (Figure 6a). We also observed the same expression pattern in E101 (Figure 6b), E109 (Figure 6c), and E115 (Figure 6d) cochlea. However, after E120 (Figure 6e), we found that the expression of JAG1 in the organ of Corti was diminished, and only a slight expression was observed in the P0 organ of Corti (Figure 6f).

Regarding the expression of DLL1, we did not detect any apparent expression of DLL1 in ATOH1-positive inner hair cells in the E87 organ of Corti (Figure 7a); however, we did observe a relatively weak expression in ATOH1-positive hair cells in the E92 cochlea (Figure 7b). Interestingly, we noticed that the expression of DLL1 in hair cells became more pronounced after E96 (Figure 7c) and was also observed in P0 (Figures 7d–h).

## 4. Discussion

Notch signaling is one of the most critical signaling pathways during development. The alleles of the *Notch* gene were first identified more than 100 years ago in a mutant *Drosophila* strain (Dexter, 1914; Morgan, 1917). Notably, Notch signaling is conserved across species from *Drosophila* and *Caenorhabditis elegans* to primates (Baron, 2003) and is widely involved in organogenesis (Siebel and Lendahl, 2017). In mammals, several sequential steps mediate the activation of Notch signaling (Bray, 2006). First, the binding of ligand proteins, such as Jagged1 (JAG1) or delta-like canonical Notch ligand 1 (DLL1), to the extracellular domain of the Notch receptor, a single-pass transmembrane receptor, induces the proteolytic cleavage of the Notch intracellular domain (NICD) by the γ-secretase complex. Activation of Notch1 is reportedly essential for the development of hair cells in the cochlea of mice (Lanford et al., 1999). In addition, the nuclear localization of NICD was observed in supporting cells of the developing cochlear at approximately embryonic day 15 (E15) (Murata et al., 2006). After cleavage of the Notch receptor, NICD is translocated into the nucleus, forming an active transcriptional complex with the DNA binding protein recombination signal binding protein for immunoglobulin kappa J region (RBPJ).

RBPJ is encoded by *RBPJ*, the mammalian homolog of the *Drosophila* gene *suppressor of hairless*. RBPJ is a transcriptional regulator important in the Notch signaling pathway (Tamura et al., 1995), which acts as an activator when bound to NOTCH proteins, whereas as a repressor when unbound (Hsieh et al., 1996). In mice, Rbpj reportedly regulates the development of pro-sensory cells in the cochlea (Yamamoto et al., 2011). This NICD-RBPJ complex binds Notch target genes and activates Notch downstream effectors, such as HES1 (Ohtsuka et al., 1999). Through these sequential steps, Notch signaling mediates two types of spatiotemporal patterning of cells during organogenesis, “lateral inhibition” and “lateral induction” (Lewis, 1998). Lateral inhibition is a process by which cells instruct adjacent cells to adopt a different fate from that of the instructing cell. In contrast, in the lateral induction process, an instructing cell induces adjacent cells to adopt the same fate as that.

In this study, we demonstrated the expression patterns of above-mentioned Notch signaling components in the developing cochlea of a primate animal model. Our study revealed several interspecies differences.

Weak activation of Notch1 has been reported in the sensory epithelium of the mouse cochlea at E13.5 (Murata et al., 2006). From E14.5 onward, with the progressing differentiation between hair and supporting cells, the activation of Notch1 has been shown to increase in supporting cells, becoming most robustly activated at E16.5 (Murata et al., 2006). Subsequently, Notch1 signaling is decreased in the cochlea after P0, with no activation of Notch1 being observed in the P7 mouse cochlea (Murata et al., 2006). Like the situation in mice, this robust activation was also observed during the differentiation of hair cells in the cochlea of the common marmoset (Figure 1). However, nuclear translocation of NOTCH1 was also detected in P0 Hensen’s cells (equivalent to P14 in mice). Compared with observations in mice, the activation of NOTCH1 was still detected in the common marmoset until relatively late in cochlear development.

Next, we investigated the expression patterns of HES1 in the developing organ of Corti of the common marmoset. In the mouse cochlea, *Hes1* was reported to be expressed in the greater epithelial ridge of the E14.5 cochlear duct (Tateya et al., 2011) and was also broadly expressed in supporting cells at E15.5 (Murata et al., 2009). However, this robust expression of *Hes1* was restricted to Hensen’s cells in the E17.5 organ of Corti (Tateya et al., 2011). Finally, no expression of *Hes1* was reported in the P0 organ of Corti (Zine et al., 2001). These previously reported expression patterns of *Hes1* were well conserved in the common marmoset (Figure 3); the expression of HES1 originated from the inside, was widely expanded, and finally restricted to Hensen’s cells. Moreover, we noticed that in the common marmoset, the changes in the expression of HES1 were like those of activated NOTCH1, as previously reported in mice (Murata et al., 2009).

The importance of the *Notch1-Hes1-Cdkn1b* axis during the developmental formation of the organ of Corti and differentiation of hair and supporting cells have been previously reported in mice, in which activation of Notch1 induces the expression of *Hes1* and the subsequent decrease and restriction in the expression of *Cdkn1b* in supporting cells (Murata et al., 2009). Combining our observations on the activation and expression of NOTCH1 and HES1, respectively, with previous observations on the expression patterns of CDKN1B in the cochlea of the common marmoset (Hosoya et al., 2022), we confirmed the observed differences between the patterns of these proteins during the differentiation of supporting cells; the expression of HES1 was observed in the E101 cochlea, whereas the expression of CDKN1B was broadly observed at later stages (at least E115) in supporting cells (Hosoya et al., 2021b). These observations implied that this axis is controlled in a more complicated manner than previously assumed in mice.

After the initial differentiation of hair cells, the activation of Notch1 and expression of Hes1 in supporting cells were observed between E14.5 and P7 in mice (approximately 11 days) (Zheng et al., 2000; Murata et al., 2006, 2009; Tateya et al., 2011). Similarly, we observed the activation of NOTCH1 and expression of HES1 in supporting cells after E87 and still observed it at P0 (equivalent to E150) in the common marmoset (at least 60 days) (Figure 5). Even considering that the cochlear development to this stage takes three times longer in the common marmoset (Hosoya et al., 2022), the activation of NOTCH1 and expression of HES1 were observed in supporting cells for a longer time than that in mice. Although the developmental importance of the prolonged-expression in these supporting cells was not determined in this study, this difference might affect the characteristics of supporting cells, especially during immaturity. Future functional studies are warranted.

To date, *Rbpj* has been reported to be ubiquitously expressed in the developing cochlea of mice and essential for the normal development of the inner ear (Yamamoto et al., 2011). In the common marmoset, we observed the ubiquitous expression of RBPJ in the developing cochlear duct up to E101 cochlea (Figure 4). However, the expression of RBPJ in the organ of Corti was subsequently reduced. This reduction was significantly preceded in hair cells and a part of supporting cells in contact with the hair cell directory, such as pillar and Deiters’ cells. Similar patterns of expression of RBPJ have been reported in several developing organs, such as the neural tube (Shi et al., 2012) and retina (Miesfeld et al., 2018). However, such non-uniform changes in the expression of *Rbpj* have not been reported during mouse cochlear development. We thus could not conclude whether these observed changes in expression in the common marmoset were interspecies-related or time-specific. However, our observations might indicate that in the later developmental stage of the primate cochlea, the activation of Notch signaling is modified or controlled not only by the expression of ligands but also by the level of expression of RBPJ. Therefore, a future functional study is required to delineate this.

The Notch ligand *Jag1* is critical for the early role of Notch signaling in the development of the cochlear pro-sensory epithelium in mice (Brooker et al., 2006; Kiernan et al., 2006). Previous studies have established that loss of *Jag1* during cochlear development resulted in the loss or substantial reduction of pro-sensory progenitor cells, leading to loss of hair cells and hearing loss (Brooker et al., 2006). In mice, *Jag1* is first expressed in the pro-sensory epithelium of the cochlea preceding the expression of *Cdkn1b* and *Sox2* (Morrison et al., 1999; Kiernan et al., 2001). At this time, *Jag1* is thought to play an essential role in the specification of the pro-sensory epithelium by inducing the expression of *Cdkn1b* and *Sox2.* By E17.5, the expression of *Jag1* is restricted to a part of supporting cells, which are located more medial than Deiters’ cells; no expression is observed in Hensen’s cells (Morrison et al., 1999; Chrysostomou et al., 2020). Subsequently, *Jag1* is continuously expressed throughout the postnatal period and adulthood (Murata et al., 2006; Oesterle et al., 2008). We previously reported that the expression of CDKN1B and SOX2 in the pro-sensory epithelium of the common marmoset was followed by the expression of JAG1, unlike that observed in mice, hence implying interspecies differences regarding the role of JAG1 in the specification of the pro-sensory domain (Hosoya et al., 2022).

Compared with previous studies, the present study examined the expression of JAG1 at a relatively late stage of cochlear development. As in mice, we observed the expression of JAG1 in a portion of supporting cells located medial to Deiters’ cells after the differentiation of hair cells (Morrison et al., 1999). This expression pattern was retained until E115 (Figure 6). However, after E120, the expression of JAG1 was decreased, and only slight expression was detected in the P0 organ of Corti. Combined with our previous observations, these results indicated that the expression of JAG1 in the Corti organ of the common marmoset cochlea had a shorter peak than that in mice.

Notch signaling is essential in limiting the number of hair cells and acquiring normal hearing through lateral inhibition. During this process, the expression of Notch ligands, such as *Dll1* (Kiernan et al., 2001, 2005), and *Jag2*, in developing hair cells and subsequent activation of Notch1 is critical for suppressing the overproduction of hair cells. In this study, we examined the expression pattern of DLL1 in the developing cochlea of the common marmoset.

In mice, the expression of *Dll1* was first observed in neonatal inner ear cells during the development of the organ of Corti from E14.5 to E15.5 (Morrison et al., 1999). It was also detected in outer hair cells at around E16.5-17.5. However, expression of Dll1 has not been observed in supporting cells during the development of the organ of Corti. Interestingly, we here detected the expression of DLL1 in hair cells in the developing cochlea of the common marmoset (Figure 7). This finding suggested that these specific expression patterns of DLL1 in hair cells of the organ of Corti are well conserved in rodents and primates and implied that the Dll1-mediated lateral inhibition of the overproduction of hair cells observed in rodents is also conserved in primates.

**FIGURE 8:**
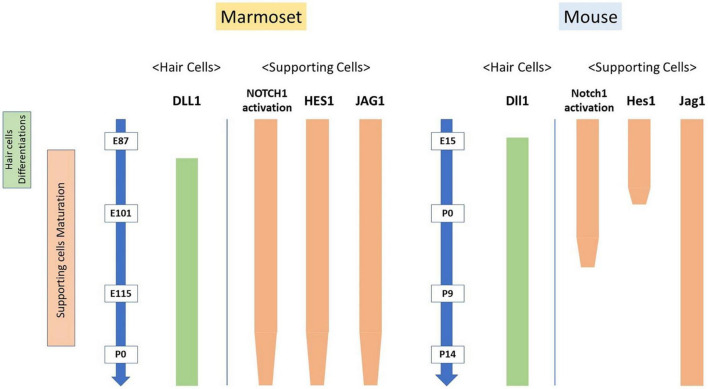
Interspecies differences in the activation of NOTCH1 and expression patterns of Notch signaling components. The expression patterns of Notch signaling components during the differentiation of hair cells are well preserved between rodents and this primate model animal. However, a relatively significant interspecies difference was observed in the maturation of supporting cells.

As shown in Figure 8, our study revealed that the expression patterns of Notch signaling components are well preserved between rodents and primates during the differentiation of hair cells. Concomitantly, we observed a relatively significant interspecies difference during the maturation of supporting cells. Combined with our previous findings (Hosoya et al., 2021b), in which we demonstrated that interspecies differences were more prominent in supporting than hair cells in the developing cochlea, our present study implied that these interspecies differences in NOTCH signaling would affect the characteristics of supporting cells. Therefore, a future functional study is needed to investigate these differences in supporting cells during these late developmental periods in this primate. Moreover, future comparative analysis of several different species analyzing the patterns or trends in the NOTCH signaling expression across species would be desired to understand the development of the hair cells. In this situation, single-cell RNA sequencing of the cochlea, which has been reported for analyzing cochlear development (Burns et al., 2015; Ranum et al., 2019; Kelley, 2022), would be useful.

We examined the developing cochlea of the common marmoset to identify similarities and differences in NOTCH signaling components between rodents and primates. Currently, the modification of Notch signaling is thought to be a feasible approach for the treatment of hearing loss from the viewpoint of regenerative therapy (Du et al., 2013, 2018; Mizutari et al., 2013; Samarajeewa et al., 2019). Our study unveiled interspecies differences between rodents and primates in the expression patterns of Notch signal components. Hence, improved knowledge of cochlear development in primates would also be helpful for future regenerative therapy strategies. However, the precise expression patterns of Notch signaling components in mice and marmosets that is more similar to humans remain unknown owing to the lack of studies exploring this signaling in humans. Therefore, we believe our study on Notch signaling in this primate model animal would serve as a foundation for future studies in humans.

We examined the developing cochlea of the common marmoset to identify similarities and differences in Notch signaling components between rodents and primates; a relatively significant interspecies difference was observed during the maturation of supporting cells. Our study on Notch signaling in this primate model animal would be helpful for future studies in humans. Moreover, as the modification of Notch signaling is thought to be a feasible approach for the potential regenerative therapy of hearing loss, improved knowledge on Notch signaling during cochlear development obtained from this primate model would also facilitate the development of future regenerative therapies for humans.

## Data availability statement

The original contributions presented in this study are included in the article/supplementary material, further inquiries can be directed to the corresponding author.

## Ethics statement

This animal study was reviewed and approved by the Animal Experiment Committee of Keio University.

## Author contributions

MH, MF, HOk, and HOz conceived and designed the experiments. MH wrote the manuscript, performed most of the experiments, and analyzed the data. All authors read and approved the final version of the manuscript.
